# Spatial proximity and scene grammar: shaping spatial representations for memory-guided actions in naturalistic environments

**DOI:** 10.1038/s41598-026-52111-8

**Published:** 2026-05-22

**Authors:** Bianca R. Baltaretu, Melissa L.-H. Võ, Katja Fiehler

**Affiliations:** 1 Department of Experimental Psychology, Justus Liebig University Giessen, Otto-Behaghel-Strasse 10F, 35394 Giessen, Hesse Germany; 2 Department of Psychology, Goethe University Frankfurt, 60323 Frankfurt, Germany; 3 Department of Psychology, Ludwig Maximilian University of Munich, 80802 Munich, Germany

**Keywords:** Spatial coding, Spatial proximity, Scene semantics, Scene perception, Memory-guided action, Virtual reality, Neuroscience, Psychology, Psychology

## Abstract

Spatial object representations are necessary for effective goal-directed actions. These representations can be produced with respect to the self (i.e., egocentrically) and/or to surrounding objects (i.e., allocentrically). For the latter, one (spatial) factor of influence is landmark proximity to target. This factor shapes allocentric representations and informs future memory-guided actions, in both impoverished and rich scenes. More recent work has suggested that cognitive factors (e.g., object semantics) can also influence allocentric coding. Here, we investigated how both spatial and cognitive factors affect allocentric coding in naturalistic environments. In a memory-guided virtual reality (VR) experiment, semantically-related targets (i.e., local objects, which are movable and manipulable, e.g. a cup) were presented on congruent or incongruent anchors (i.e., large, immovable objects, predictive of local object locations). After an imperceptible anchor shift (or not), a target had to be placed in its remembered position. Placement performance results indicated that anchors were, indeed, used to build up spatial target representations. Additionally, these representations showed strong effects of proximity-to-anchor, with no differential effects of phrase-level semantic congruency. Taken together, in our memory-guided task, we found that spatial proximity plays a dominant role in allocentric coding in naturalistic (virtual) scenes.

**Supplementary Information:**

The online version contains supplementary material available at 10.1038/s41598-026-52111-8.

## Introduction

Effective interaction with the objects in our environment requires accurate and precise spatial representations of where they are located. This can be accomplished using an egocentric reference frame, where an object’s position is represented with respect to the self (i.e., to the eye, hand, etc.)^1–5^ or using an allocentric reference frame, where object positions are related to other, surrounding objects^1–4,6–8^. In terms of allocentric coding, both spatial and cognitive factors have separately been shown to influence these representations^9^. Spatial factors, such as proximity-to-landmark, contribute to allocentric coding with landmarks closer to targets exerting a stronger influence compared to landmarks further away^10^. This has been shown in both simplistic scenes with abstract objects^10^, as well as in more naturalistic, 2D images^11^. Cognitive factors, such as object semantics, have also been shown to influence allocentric coding. In a virtual reality (VR) memory-guided reach task conducted in a naturalistic environment, the spatial representation of a target object’s position was influenced more by surrounding objects of the same semantic category (e.g., the banana’s position relative to other fruits), as opposed to a different semantic category (e.g., the banana’s position relative to office objects)^12^. Importantly, these studies have been conducted on objects of the same class type (e.g., surrounding dots on target dot^10^ or surrounding small, moveable objects on another small, moveable target object^12^, but rarely on different object types, such as those that determine a room’s composition (e.g., a fridge)^13–15^.

The representational organization of these different object types, according to the scene grammar framework^16,17^, is suggested to follow a hierarchy. At the lowest level of the scene grammar hierarchy, scenes contain smaller, so-called ‘local’ objects that tend to serve as the targets of our actions (e.g., the coffee cup we pick up or the spoon with which we stir). The identity and location of these local objects are predicted by the larger, typically immovable objects that occupy everyday spaces, i.e. anchors, such as stoves or showers or couches, which represent the second level of the hierarchy. Often, we spatially associate local objects and anchors in a functionally meaningful manner (e.g., the espresso cooker on top of the stove); these meaningful and functionally related clusters are known as ‘phrases’, which then again form the scene itself (a kitchen, an office, etc.). Typically, objects that serve as action targets are considered ‘local objects’ in the scene grammar framework, as they are smaller and easy to move around. Given that these cognitive factors (scene grammar) influence our behaviour in everyday environments, e.g. by directing attention, guiding visual search, and improving memory representations^17–25^, one would assume that they also play a role for goal-directed actions in naturalistic environments.

To determine which factors influence spatial representations, shift paradigms are often used, where local objects in the surroundings are imperceptibly shifted (or not) and the remembered target location is assessed as a function of those local object shifts^2,4,6,8,12–14,26–30^. While surrounding local objects have been shown to influence the allocentric coding of other local objects for memory-guided actions^12^, another study found a significant influence of anchors on the positioning of local objects^31^, though no additional semantic (phrase-level) effects were found^31^. Given that, in our everyday environments the physical proximity of local objects to their semantically related anchors varies continuously due to using and therefore moving objects^16,17,19,22^, it is important to understand how spatial and cognitive factors contribute to allocentric coding for memory-guided actions.

To address the influence of anchors on spatial representations of local objects in naturalistic environments and how these are modulated by spatial (proximity-to-anchor) and cognitive (scene grammar) factors, we used a similar experimental design to Baltaretu et al.^31^. The experiment began with the initial viewing of the scene, wherein three local objects (all of the same semantic category) were presented in or on an anchor – either semantically congruent (Phrase-congruent condition; e.g., stove objects on the stove) or incongruent (Phrase-incongruent condition; e.g., stove objects in the fridge). After this, the scene was presented again without the local objects, but either with the anchors in their original locations (No-shift condition) or one of the anchors shifted imperceptibly (Shift condition). After a short period of time, one of the local objects appeared in front of the participant, who then had to grab this object and walk to place it back in its remembered location. We had three main hypotheses, one related to the use of anchors for allocentric representations and another set related to the effects of spatial and cognitive factors on this process. For the *Anchor Shift Hypothesis*, we anticipated that placement behaviour would show strong effects of anchor shifts on remembered local object representations^31^. For the *Phrase-level Semantics Hypothesis*, we anticipated placement behaviour toward local objects to be more strongly influenced by phrase-congruent versus phrase-incongruent anchors. Finally, for the *Anchor Proximity Hypothesis*, we anticipated placement behaviour to be more strongly affected by anchors close in spatial proximity compared to those far away. In addition, we explored a potential interaction effect between phrase-level semantics and anchor proximity. To preview, we found that allocentric representations of local objects *are* influenced by anchors in naturalistic environments. In particular, proximity-to-anchor affected allocentric coding, whereas phrase-level semantic congruence showed no effect.

## Methods

### Participants

Using an a priori power analysis based on Karimpur et al.^12^ (effect size: 0.36; alpha: 0.05; correlation: 0.49; desired minimum power: 0.95), thirty participants between the ages of 18 and 35 (26.9 years of age +/- 4.06) years completed the experiment. Participants were all right-handed as assessed by the Edinburgh Handedness Inventory (EHI; 89.4 +/- 20.86). They provided informed consent and were remunerated for their time. Experimental protocols were in concordance with the general principles of the Declaration of Helsinki (2013; without preregistration of the study) and approved by the local ethics committee (Department of Psychology) at Justus-Liebig-University Giessen (Germany).

### Apparatus

All details are the same as in Baltaretu et al.^31^ except for hardware (i.e., ASUS computer, 13th Gen Intel(R) Core™ i9-13900 K processor, 64 GB RAM, with an NVIDIA GeForce RTX 4090 graphics processor). We used the HTC Vive Pro Eye VR head-mounted display (HMD; HTC Corporation, Xindian, New Taipei, Taiwan), with SteamVR (v.2.9.6) and Unity (v.2021.3.5f1, Unity Technologies, Inc., San Francisco, CA, USA) to present stimuli. Four lighthouse base stations were used to record HMD and controller positions throughout the experiment. (While eye movements were also recorded, these were not analyzed, nor reported here.)

### Set-up and stimuli

**Fig. 1:**
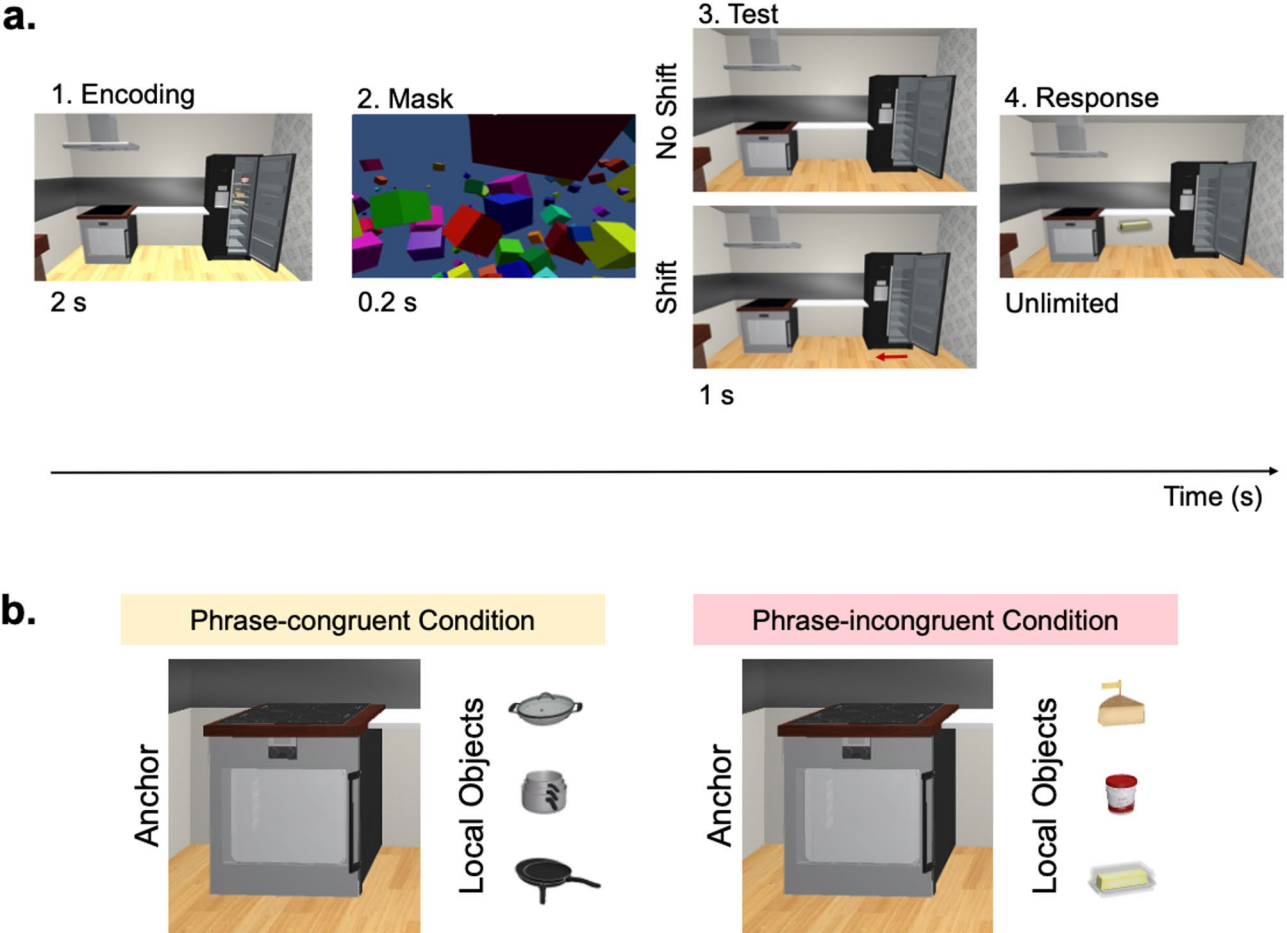
Experimental VR paradigm, phrase conditions and object types. **a** A typical trial had four main events: (1) Encoding (2 s), (2) Mask (0.2 s), (3) Test (1 s), and (4) Response (unlimited). During Encoding, a subset of three targets was presented physically interacting with an anchor (i.e., on top of or inside an anchor; here: congruent), followed by a 3D mask. During the Test event, all targets were absent and both anchors were presented in their Encoding event locations (No-shift condition) or one of the anchors was shifted leftward or rightward by 0.075 m (Shift condition). The last, Response event occurred, during which one of the three targets appeared in front of the participant that had to be re-placed in its remembered position. **b** Depicted are the combinations of different object types, an example anchor and the local objects, according to phrase semantics. In the Phrase-congruent condition, we have the stove as the example anchor along with its phrase-related local objects (upper to lower: wok pan, stacked pots, and frying pans). In the Phrase-incongruent condition, we tested the relationship between stove and its phrase-unrelated local objects (upper to lower: cheese, yogurt, and stick of butter). This was done for both anchors on either side of the central neutral shelf, for all four scene types (two kitchens and two bathrooms).

Here, we used the same scenes as in Baltaretu et al.^31^ presenting two kitchen and two bathroom scenes. Each of the scenes depicts two anchors (e.g., stove and fridge on either side of a central shelf; Fig. 1). The same sets of three-object sets were used as in Baltaretu et al.,^31^ with one important difference: Here, we presented each set of stimuli either in or on their congruent or incongruent anchors (e.g., stove objects on stove or stove objects in fridge, respectively). In addition, we took advantage of the ability to test two levels of the scene grammar hierarchy, not previously tested on spatial coding, by (1) presenting target objects with their congruent anchors (Phrase-congruent condition) or incongruent anchors (Phrase-incongruent condition) and (2) modulating the distance of the congruent anchor (Anchor-congruent condition) or the incongruent anchor (Anchor-incongruent condition). This resulted in four conditions: (1) Phrase-congruent, Proximal-anchor, (2) Phrase-congruent, Distal-anchor, (3) Phrase-incongruent, Proximal-anchor, and (4) Phrase-incongruent, Distal-anchor. This allowed us to test how proximity-to-anchor is used when rich scene semantic information is available for spatial coding. Moreover, introducing Phrase-incongruent conditions, where target and anchor objects were positioned in a semantically-incongruent manner, provided the opportunity to test how proximity and scene grammar interact to shape allocentric spatial coding.

In terms of target placement, objects were placed in a pseudorandomized way such that (1) objects were not directly at the edges of the anchor and (2) object occlusion was minimized and/or did not occur. In each trial, one set of targets was presented according to the semantic condition type (e.g., Phrase-congruent or -incongruent). Across the four scenes, with two anchors per scene, and a specific target subset per anchor, this resulted in 24 targets used for the entire experiment.

### Procedure

We used the same procedure and experimental paradigm as in Baltaretu et al.^31^, which is outlined as follows. Before the experiment, participants were given the opportunity to complete twelve baseline trials to familiarize themselves with the task. The trial sequence was the same as described next and was performed in a kitchen and bathroom with target objects that were never presented in the experimental trials.

At the beginning of each trial, a pair of virtual blue shoes appeared on the virtual floor, indicating the starting position. The starting position was the same within each scene (and across all participants), aligned with the centre of the shelf (equidistant between the two anchors), and located 0.5 m in front of middle of the shelf (~ 1.2 m to the centre of either flanking anchor). Participants were required to stand on the blue shoes and then, press the trigger button on the controller to commence the trial.

Each trial comprised four main phases (Fig. 1): (1) Encoding, (2) Mask, (3) Test, and (4) Response. The first, Encoding phase lasted 2 s, during which time a scene was presented with target objects interacting with an anchor based on the condition type (see above for the four main semantic conditions). After this, a 0.2 s 3D mask was presented to prevent afterimage effects. The Test phase followed and lasted for 1 s, which was when the same scene was presented again with (1) no targets present and (2) the two anchors in their Encoding positions (No-shift condition) or one of the anchors was shifted imperceptibly (Shift condition) by 0.075 m^31,32^ leftward or rightward, unnoticeably to the participant. Lastly, during the Response phase, one of the three targets appeared approximately 0.3 m at eye level in front of the participant, who then had to reach out with the controller and grab the object (using the two side buttons) and walk to place it back in its remembered position. Once they were satisfied with the position, participants pressed the trigger button to end the trial and find the pair of blue shoes for the starting position of the next trial.

This procedure occurred for each of the three targets in each anchor-related target subsets, in the two semantic conditions (Phrase-congruent and -incongruent) and two proximity conditions (Proximal (anchor close to local objects) and Distal (anchor far from local objects)), for each of the four scenes, and for each shift condition (No-shift, Left Shift, and Right Shift). This resulted in a total of 240 trials per participant (48 No-shift; 24 Phrase-congruent, Proximal-anchor, Left Shift; 24 Phrase-congruent, Proximal-anchor, Right Shift; 24 Phrase-congruent, Distal-anchor, Left Shift; 24 Phrase-congruent, Distal-anchor, Right Shift; 24 Phrase-incongruent, Proximal-anchor, Left Shift; 24 Phrase-incongruent, Proximal-anchor, Right Shift; 24 Phrase-incongruent, Distal-anchor, Left Shift; and 24 Phrase-incongruent, Distal-anchor, Right Shift) - these were randomly split into 60 trials per block (four blocks in total) in order to make the experiment more manageable for participants. In total, the experiment took approximately one hour to complete.

### Quantification and statistical analysis

#### Placement behaviour data

The last position of the object, prior to trial termination, was recorded and used for further analysis. In this experiment, the shift of anchors occurred along the X-axis in the virtual environment and, as such, we expected systematic placement errors mainly along this axis only. Therefore, we restricted the calculation of placement errors to the x-axis. First, we calculated the placement error as a way of determining the influence of anchor shift modulations on the remembered target location. To do this, for each target in a given scene and Phrase condition, we obtained the X-coordinate in the Response phase for the Shift and No-shift conditions. We subtracted the values in the No-shift condition from the corresponding values in the Shift-condition, matched for target and semantic condition for each participant (e.g., X-coordinate for the yogurt in the Phrase-congruent condition in Kitchen 1 when the fridge was shifted (Shift condition) minus X-coordinate for the yogurt in the Phrase-congruent condition in Kitchen 1 when the fridge was not shifted (No-shift condition)). Given that placement errors are directional, negative errors indicate leftward placement of the target relative to its placement in the No-Shift condition, whereas positive errors indicate rightward placement of the target relative to its placement in the No-Shift condition. As such, of the 7200 trials across all 30 participants, we analyzed the remaining 5760 Shift trials (192 trials per each of the eight condition types – e.g., Phrase-congruent, Proximal-anchor, Left Shift, etc.). Due to a meta-labeling error, we excluded one full scene from further analyses (no effect on the statistical power of the study), resulting in 4320 trials (144 trials per each of the eight condition types). We, then, excluded trials with placement errors + / - three standard deviations (SDs) away from the mean (i.e., 49 trials in total: (1) 21 Left Shift trials and 28 Right Shift trials and, in terms of semantic condition, (2) 10 Phrase-congruent, Proximal-anchor trials, 13 Phrase-congruent, Distal-anchor trials, 13 Phrase-incongruent, Proximal-anchor trials, and 13 Phrase-incongruent, Distal-anchor trials).

To the remaining 4271 trials, we applied linear mixed-effects modeling (LMM) to the placement errors to address the Anchor Shift Hypothesis. Trial-level models included Anchor Shift as a fixed effect and combinations of random intercepts to account for variability due to participants, targets, and scenes.

### Allocentric weights

To test the Phrase-level Semantics Hypothesis and the Anchor Proximity Hypothesis, we applied LMMs to the allocentric weights. Trial-level models including fixed effects of Phrase-level Semantics (Congruent, Incongruent) and Proximity (Proximal, Distal), as well as their interaction, were applied to the data. We also tested and compared model fits with random intercepts for participants, targets, and scenes, as well as all possible combinations thereof to account for potential variability in the data.

Model fits were determined using the Akaike Information Criterion (AIC) score and delta AIC values. F-values, p-values, and partial eta-squared (η^2^_p_) values are provided for each model (i.e., for placement errors and allocentric weights). An alpha-level of 0.05 was used to determine statistical significance. Wherever appropriate, Bayes factor results (BF_10_) are also provided as a way of quantifying evidence for the absence of effects.

All statistical tests were conducted using R (v4.5.3).

## Results

### Anchors influence allocentric coding in naturalistic scenes

**Fig. 2:**
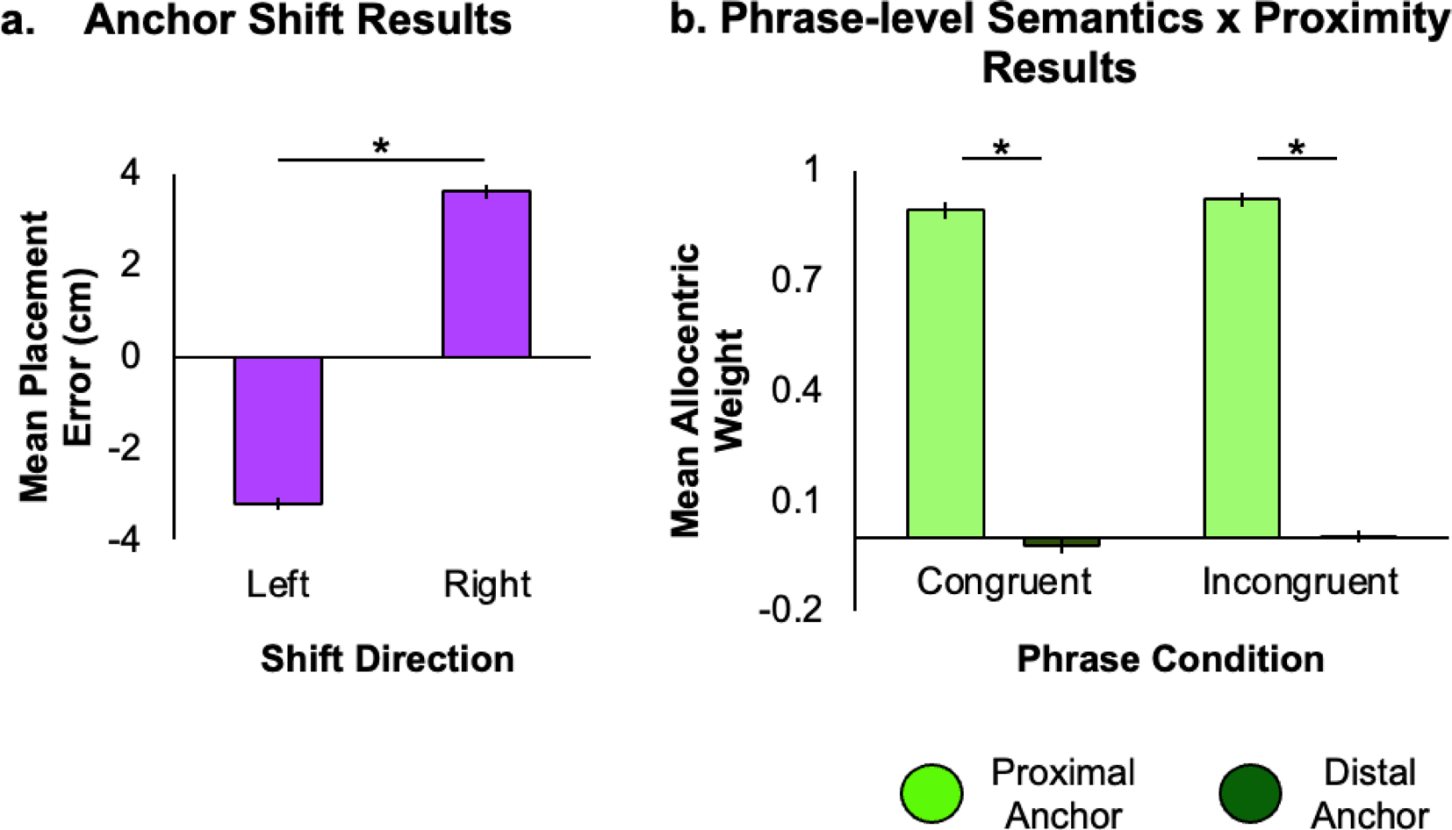
Mean placement behaviour results in testing the three hypotheses. (**a**) Mean placement errors (cm; with standard error of the mean, SEM, as error bars) presented for the shift conditions (Left and Right Shifts). There is a significant difference between the effects of anchor shifts on the remembered target position (i.e., leftward, negative placement errors of targets found for leftward anchor shifts, and vice versa). (**b**) Mean allocentric weights (errors bars are SEMs) presented for the proximity conditions (Proximal, lime green, and Distal, hunter green) within each of the phrase (Congruent, Con, and Incongruent, Incon) conditions. There is no significant interaction between the two factors, nor a significant difference between the effects of phrase-congruent and -incongruent anchor shifts on the remembered target position. However, there is a significant difference between the proximity anchor conditions. Namely, there are large effects of Proximal anchor shifts, and virtually no effects of Distal anchor shifts, on the remembered target position. *** *p* < 0.001.

To test the first hypothesis, we applied linear mixed-effects modeling to the placement errors. Model comparisons indicated that the best LMM included Anchor Shift as a fixed effect and a modest contribution of target variability as a random intercept (Table S1; lowest AIC and delta AIC). From this, we found that placement errors were significantly affected by anchor shifts (F(1, 4240.5)_Anchor Shift_ = 1163.8, *p* < 2.2 × 10^− 16^, η^2^_p_ = 0.22; Fig. 2a). In concordance with previous results^31^, we confirm that anchors play an important role in the spatial coding of local objects. These findings suggest that naturalistic environments with rich scene grammar contain robust sources of allocentric landmarks to produce and update spatial representations of target positions for memory-guided actions.

### Anchor proximity modulations differentially affect allocentric coding of local object targets

To test our Phrase-level and Proximity hypotheses, we applied LMMs to the allocentric weights. Model comparisons suggested that the best LMM included fixed effects of Phrase-level Semantics and Proximity, as well as with their interaction, with no improvement in fit when including random intercepts for participants, targets and / or scene. In this model, we did not find a significant main effect of phrase-level semantics on allocentric coding (F(1, 4267)_Phrase-level Semantics_ = 1.424, *p* = 0.233, η^2^_p_ = 3.34 × 10^− 4^; BF_10_ = 0.057 (strong evidence for the absence of a main effect of Phrase-level Semantics); Fig. 2b). Consistent with the Anchor Proximity Hypothesis, in which we hypothesized that shifts of Proximal vs. Distal anchors would result in larger allocentric weights, we did find a significant main effect (F(1, 4267)_Anchor Proximity_ = 1677.59, *p* < 2.2 × 10^–16^, η^2^_p_ = 0.28). Lastly, we found no significant interaction between phrase-level semantics and proximity-to-anchor (F(1, 4267) = 0.014, *p* = 0.907, η^2^_p_ = 3.32 × 10^− 6^; BF_10_ = 0.048 (strong evidence for the absence of a Phrase-level Semantics and Anchor Proximity interaction). We also performed post hoc t-tests against zero (i.e., in the No-shift condition) for the Distal conditions and found that neither is significantly different from an allocentric weight of zero (t(29)_Phrase-congruent Distal_ = -1.08, *p* = 0.287, Cohen’s d = 0.20; BF_10_ = 0.33 (evidence in favour of the null hypothesis (i.e., allocentric weight is not different from zero)); t(29)_Phrase-incongruent Distal_ = 0.26, *p* = 0.797, Cohen’s d = 0.05; BF_10_ = 0.20 (evidence in favour of the null hypothesis (i.e., allocentric weight is not different from zero))). Finally, we performed a post hoc exploratory analysis of the placement errors between the Response and Encoding to determine if phrase semantics might affect memory precision. In doing so, we found no significant difference with respect to placement error memory accuracy (t(29) = − 0.06, *p* = 0.951, Cohen’s d = 0.01, BF_10_ = 0.19 (moderate evidence in favour of the null hypothesis); Mean_Phrase-congruent_ = -0.002 cm, SE_Phrase-congruent_ = 0.005 cm; Mean_Phrase-incongruent_ = -0.002 cm, SE_Phrase-incongruent_ = 0.002 cm). When these errors were tested against zero, we also found no baseline differences in memory accuracy in either semantic condition in this experiment (t(29)_Phrase-congruent_ = -0.41; *p* = 0.684, Cohen’s d = 0.08, BF_10_ = 0.21 (moderate evidence in favour of the null hypothesis); t(29)_Phrase-incongruent_ = -0.77; *p* = 0.449, Cohen’s d = 0.14, BF_10_ = 0.25 (moderate evidence in favour of the null hypothesis)). Overall, these findings suggest that, given our particular manipulations and experimental setup, when spatial and cognitive factors are both modulated in a memory-guided task, spatial factors (proximity-to-anchor) might already affect allocentric coding to such an extent that more cognitive factors do not additionally modulate behaviour.

## Discussion

In this experiment, we set out to test how different factors (spatial vs. cognitive) may exert effects on allocentric coding of local target objects in naturalistic scenes. Our results suggest that anchors may act as landmarks for the establishment and updating of spatial representations of local object targets (Fig. 2a). Moreover, spatial proximity-to-anchor affected allocentric coding, while phrase-level semantic congruency did not show an influence in this task. These findings suggest that allocentric coding of local objects in memory-guided tasks may be particularly sensitive to spatial factors, while more subtle, cognitive factors might exhibit effects under conditions that remain to be uncovered.

### The role of scene context for allocentric representations

A major component to carrying out goal-directed actions is knowing where objects are in our surroundings. In minimal environments, devoid of context apart from screen edges and a monochromatic background, much work has been conducted to identify how object locations are determined under various circumstances. While we are able to reference, say a black dot on a large gray screen, egocentrically, kinematic measures (movement accuracy and variability) seem to improve with contextual cues^33–36^. Under conditions where view of the action target is no longer possible or some delay is introduced between viewing the target and an action to indicate its remembered location, egocentric coding is still sufficient, but errors (or deviations) emerge^4,33–37^. When additional, contextual cues (i.e., other dots or crosshair landmarks) are provided, they allow for object-to-object relations to be used in representing the spatial layout of the environment and the target’s position therein^14,38^. These relationships, in allocentric referencing, help to mitigate errors that creep in when egocentric information is no longer sufficient in memory-guided actions^37^. These two codes exist and even work in tandem to facilitate movement planning under both visually- and memory-guided conditions^28,30,32,34–36^.

Despite the importance of research using such reduced stimuli, novel approaches are needed to capture the complexities of real-world environments. One previous attempt to transcend into more complex scene space was a task that was able to compare the accuracy of target representation when a single target was presented against a monochromatic background or a schematized landscape background^38^. After a delay of several seconds, accuracy in the presence of the landscape was maintained, while egocentric-related accuracy decreased nearly instantly^39^. Another important step toward understanding spatial coding in realistic, yet experimentally controlled, settings can be achieved in VR experiments, using 3D natural scenes and stimuli.^2,8,13,30,40^ With such VR experiments, it was found that representations of objects reflected a greater weighting of object-object spatial relationships when the target and surrounding objects belong to the same semantic category^40^. This suggests that allocentric object coding does not show equal weighting amongst all object-object relationships, highlighting a noteworthy difference to previous, more simplistic experiments.

Moreover, we showed, just like the simpler dot in the vicinity of a crosshair landmark^14^, that local objects can be related to other, special objects, such as anchors in realistic (virtual) environments^31^. Akin to the dot-crosshair relationship, the large-scale structure of a scene (i.e., the layout of the anchors and targets) is suggested to be important for establishing and updating allocentric representations of targets^31^. Here, we made use of that relationship and tested how spatial coding is affected by phrase semantics. We found that, under the specific task demands of our experiment (e.g., no search required), allocentric weights were similar across the two phrase conditions (Fig. 2b). There are many possible explanations for this, which include the strength of spatial proximity and semantics manipulations, how these two lined up with the response demands, and sensitivity to often more subtle, semantic effects. It could also have been that semantics play a role in baseline memory accuracy - while we did not find such differences in this experiment, this is important to address in future experiments. In addition, the brief Encoding phase of only two seconds (just as in Baltaretu et al.^31^) may have contributed to the lack of this effect here^41,42^. Given that elsewhere, transformations from an allocentric encoding to an egocentric motor one have been found to be computationally demanding^43^, it is important to identify whether this may have influenced the possibility of assessing semantic influence in the Encoding phase and / or whether this additionally contributed to the motor response. Although our analyses likely accounted for motor noise introduced from the Encoding to Reponses phases (i.e., by subtracting condition-specific No-shift conditions), future computational approaches and investigations should aim to unpack the delicate balance between scene complexity, cognitive demand, task switching, motor noise and how these contribute to allocentric coding in naturalistic (virtual) memory-guided tasks. Lastly, while we did not differentiate specifically between an object’s purpose or function, this is a point of importance to address in such naturalistic designs. It has been shown that these differences do appear in how the brain is engaged (functional vs. semantic relevance) for spatial coding^44,45^, but it is not known how this may have contributed to the Phrase-level semantic manipulations made here. In this naturalistic, memory-guided task, our findings imply that our perceptual and spatial coding machinery is well-suited to adapt landmark-selection strategies to our complex environments (i.e., using anchors, which may reflect more robust landmarks than local objects), even when the statistical regularities that we have learned are violated (i.e., across phrase conditions). While we did not find evidence in support of phrase-level semantics here, this may occur under other experimental conditions or tasks, which should be further explored. Future investigations should characterize the contributions of the multitude of (relevant) factors that characterize more naturalistic environments and how they may influence spatial coding in memory-guided tasks.

### The role of spatial proximity for landmark selection

Beyond scene context, other factors, such as the distance between target and landmarks^10,39^, can also influence allocentric coding. Using experiments with simplistic stimuli and scenes, kinematic measures, such as reach endpoints, for surrounding landmarks were found to reflect that proximal landmark use leads to less endpoint variability than for distal landmarks^46–48^. This suggests that landmarks close to a target act to mitigate noise for the eventual reach behaviour, offering a reliable signal to weigh for allocentric coding^10,14^. In a previous study using photos of real-world scenes, participants were able to choose the most appropriate landmark in order to identify a designated target al.locentrically^11^. However, this was limited to landmarks located up to 10° away from the target, suggestive of a large, yet limited spatial range of use^11^. How does proximity of landmark-to-local-object influence memory-guided actions in 3D environments?

In our VR task, participants were asked to recall and reposition the local target object in its remembered position. They were given no instructions about which landmarks to use to accomplish this task. We essentially found that only proximal anchors shaped allocentric representations of local target objects, while distal anchors (over 70° away from centre to centre) had no effect whatsoever (Fig. 2b). This proximity effect resulted in allocentric weights that greatly outweigh those found in previous memory-guided experiments, in which local object – local object spatial relationships were being probed (~ 90% vs. ~50%, respectively)^2,8,13,31^. Given the difference in weighting of allocentric information due to landmarks (anchors vs. surrounding local objects) on local target objects, is it possible that there is a hierarchy of reliability for different object types (local vs. anchor objects) when it comes to choosing a landmark – do anchors come first and are then followed by local objects (with a stronger weighting of closer versus more distant local objects)? Moreover, naturalistic scenes can contain additional landmarks that may also contribute to the spatial representations of local objects in the environments, including walls and overall room layout^49–51^. Having multiple landmarks present has been shown to influence the use of allocentric (over egocentric) representations^52^ and for this reason, among others, one possibility is that we instinctively choose which landmarks to have in a given scene and where they should be positioned. Namely, we organize our environments in a way that supports our actions^19^ in a way that renders us more effective in accomplishing our goals. However, our evolving understanding of how to optimize the layout of our surroundings and in which ways this contributes to spatial coding in memory-guided actions in naturalistic scenes requires further systematic assessments.

In sum, in this VR experiment, we modulated the scene layout to test proximity-to-anchor and phrase-level semantics effects on allocentric coding of local objects in a memory-guided placement task. Allocentric coding of local objects was influenced by proximal rather than distal anchors, with similar effects across semantic (phrase) modulations. These findings suggest that, in rich, naturalistic scenes, spatial coding for goal-directed actions is differentially influenced by spatial and cognitive factors that change across the scene grammar hierarchy.

## Supplementary Information

Below is the link to the electronic supplementary material.

Supplementary Material 1

## Data Availability

Data can be accessed at the following link (https://osf.io/5us4b/overview? view\_only=4532d536138745ba914f789a2841be2e) and / or upon request from Bianca R. Baltaretu (b.baltaretu@gmail.com).
