# Supplementary material for: Spatial proximity and scene grammar: shaping spatial representations for memory-guided actions in naturalistic environments
Source: Sci Rep. 2026 May 22;16:15982. doi: 10.1038/s41598-026-52111-8 (PMC13195109; doi:10.1038/s41598-026-52111-8)
Supplement: Supplementary file 1 — Supplementary Material 1 [file 41598_2026_52111_MOESM1_ESM.docx]

**Supplementary Materials**

**Table S1. Linear mixed-effect model comparisons of random intercepts for participants (part), target, and scene for placement errors. Included are the random effects (intercepts) tested and the corresponding AIC and delta AIC values.**

**Table S2. Linear mixed-effect model comparisons of random intercepts for participants (part), target, and scene for allocentric weights. Included are the random effects (intercepts) tested and the corresponding AIC and delta AIC values.**
